# PFAS Exposure and Metabolic Disorders: Mechanistic Insights into Lipid and Glucose Homeostasis

**DOI:** 10.3390/biom16071056

**Published:** 2026-07-19

**Authors:** Xinyi Chen, Weijing Wen, Simeng Gu, Fanjia Guo, Zhe Mo, Zhijian Chen, Sujun Yan, Xiaofeng Wang

**Affiliations:** 1 Department of Environmental Health, Zhejiang Provincial Center for Disease Control and Prevention, 3399 Bin Sheng Road, Binjiang District, Hangzhou 310051, China; 2511140074@nbu.edu.cn (X.C.);; 2 School of Public Health, Health Science Center, Ningbo University, Ningbo 315211, China

**Keywords:** PFAS exposure, lipid homeostasis, glucose homeostasis, obesity

## Abstract

Per- and polyfluoroalkyl substances (PFAS) are a class of synthetic fluorinated chemicals characterized by high environmental persistence and widespread occurrence, which have attracted increasing attention due to their potential adverse effects on human health. As the global burden of chronic diseases related to lipid and glucose dysregulation rises, identifying environmental contributors is increasingly important. Growing evidence links PFAS exposure to metabolic disorders, particularly those involving lipid and glucose metabolism. This review summarizes current findings on the mechanisms by which PFAS disrupt metabolic balance, with a focus on pathways involved in fatty acid uptake and oxidation, nuclear receptor signaling, oxidative stress, inflammation and insulin signaling. Furthermore, we highlight the convergence of molecular pathways involved in PFAS-induced lipid and glucose metabolic alterations, which may provide a mechanistic basis for understanding the development of metabolic disorders. Finally, we discuss current research limitations and future perspectives, including the potential application of computational approaches and emerging technologies to further elucidate PFAS-related metabolic effects. A comprehensive understanding of these mechanisms may help identify targets for the prevention and treatment of metabolic diseases, including obesity and type 2 diabetes, and provide regulatory policies on PFAS.

## 1. Introduction

Per- and polyfluoroalkyl substances (PFAS) are defined as fluorinated substances that contain at least one fully fluorinated methyl or methylene carbon atom (without any H/Cl/Br/I atom attached to it) [1]. According to different chemical structures, PFAS can be divided into perfluoroalkyl substances (including PFOA and PFOS) and polyfluoroalkyl substances (including FTOHs and FOSAs) [2,3,4,5]. The main PFAS categories and representative substances covered in this review are summarized in Table 1. As common fluorinated amphiphiles, PFAS possess a unique molecular structure: a hydrophilic polar head group and a hydrophobic and oleophobic perfluorinated tail. This gives them exceptional stability, surface activity, and resistance to both water and oils [6,7]. Thus, they have been utilized in various industrial and commercial applications, including surfactants, fire-fighting foams, and stain-resistant coatings, since the onset of production in the late 1940s [8,9]. Their extensive use and environmental persistence have led to their widespread environmental accumulation, posing serious ecological risks and significantly elevating exposure levels for both humans and animals. Therefore, PFAS manufacturing has shifted toward new alternatives, such as the short-chain analogs perfluorobutyric acid (PFBA) and perfluorobutanesulfonic acid (PFBS), as well as chlorinated polyfluoroether sulfonates (e.g., Cl-PFESA, trade name F-53B) [10,11]. While alternatives were developed to replace hazardous long-chain PFAS, their health risks are now gradually being uncovered [12]. Some PFAS can enter and accumulate in the human body through food, water, and other environmental sources, with detectable concentrations commonly found in blood, liver, and other protein-rich tissues [13,14,15,16]. Acting as endocrine-disrupting chemicals, accumulated PFAS are increasingly implicated in disrupting metabolic homeostasis [17].

Metabolic disorders such as type 2 diabetes mellitus (T2DM) and obesity represent a major global health challenge, driven by their escalating prevalence, morbidity, and mortality, with disrupted homeostasis of glucose and lipid metabolism playing a significant role. As a chronic and progressive disease, obesity has seen its worldwide prevalence nearly triple since the mid-1970s [18,19,20,21,22]. If current trends persist, over half of the global population is projected to be overweight or obese by 2035, imposing a substantial economic burden [23]. The burden and impact of T2DM on a global scale are far from insignificant. The International Diabetes Federation (IDF) estimated that the prevalence of diabetes mellitus among adults aged 20–79 years was approximately 9.8% (536.6 million adults) in 2021 [24]. Furthermore, excessive fat accumulation is one of the causes of T2DM, and the risk of T2DM increases linearly with BMI [17,18]. Meanwhile, the comorbidity rate of obesity among T2DM patients remains high. For example, a systematic review including 57 studies reported a comorbidity rate of 75.27% in children [25].

Recent research has increasingly focused on environmental pollutants, particularly PFAS, as endocrine-disrupting chemicals influencing obesity, T2DM, and related metabolic conditions [26,27]. PFAS disturb key metabolic regulatory networks by interfering with enzyme activities, receptor functions, and signaling pathways involved in glucose and lipid metabolism. This disruption impairs the synthesis, breakdown, and transport of glucose and lipids, ultimately disrupting metabolic homeostasis [28,29]. Obesity and T2DM are closely linked through shared pathological mechanisms, including ectopic lipid accumulation, chronic low-grade inflammation, and insulin resistance in key metabolic tissues such as the liver, skeletal muscle, and white adipose tissue (WAT) [30]. These insights underscore the systemic and interconnected nature of glucose and lipid metabolism regulation. Here, this review focuses on obesity and T2DM as key metabolic endpoints, synthesizes current evidence on the molecular mechanisms through which PFAS disrupt glucose and lipid metabolism, compares differences among individual PFAS, and delineates how these disrupted pathways interconnect to guide future research in environmental metabolic health.

## 2. PFAS Exposure Induces Lipid Metabolic Disorders

### 2.1. Lipid-Related Epidemiological Evidence

Dyslipidemia, characterized by abnormal circulating levels of total cholesterol (TC), low-density lipoprotein cholesterol (LDL-C), high-density lipoprotein cholesterol (HDL-C), and triglycerides (TGs), represents one of the most commonly reported metabolic outcomes associated with PFAS exposure [31]. Given the increasing global burden of obesity and other lipid-related metabolic disorders, numerous epidemiological studies have investigated the potential association between PFAS exposure and dyslipidemia. Large-scale cross-sectional studies and meta-analyses have consistently demonstrated positive associations between serum PFAS levels and TC, with relatively consistent positive associations also reported for LDL-C. In contrast, associations with TGs and HDL-C remain inconsistent across studies [32,33]. Similar associations have also been reported among adolescents as well as in studies examining prenatal PFAS exposure and adverse metabolic outcomes in offspring [34]. Compared with cross-sectional studies, longitudinal cohort studies provide stronger temporal evidence supporting a potential role of PFAS exposure in the development of metabolic disorders [35,36,37].

Although the association between PFAS exposure and dyslipidemia, particularly elevated TC, has been reproduced across multiple epidemiological studies, the evidence for obesity is considerably less consistent. Several prospective cohorts have reported positive associations between prenatal or lifelong PFAS exposure and increased adiposity, BMI, or waist circumference, whereas other studies have observed weak, null, or even inverse associations. These discrepancies suggest that the obesogenic effects of PFAS may depend on PFAS congeners, exposure windows, sex, developmental stage, and background metabolic status rather than representing a universal response [38,39].

The heterogeneity of epidemiological findings likely reflects multiple sources of variation rather than the absence of biological effects. Different PFAS congeners possess distinct toxicokinetic characteristics, receptor-binding affinities, and elimination half-lives, while study populations differ substantially in age, sex, baseline metabolic status, co-exposure patterns, and exposure levels. Furthermore, the use of different metabolic endpoints and study designs may further contribute to inconsistent findings across studies. Despite these variations, the growing body of mechanistic evidence has consistently demonstrated that PFAS target several highly conserved pathways regulating lipid homeostasis, providing important biological plausibility for the observed epidemiological associations [39,40].

Longitudinal studies and epidemiological evidence suggest that PFAS exposure is associated with lipid metabolism disorders, including obesity and potentially non-alcoholic fatty liver disease (NAFLD), although direct evidence linking serum PFAS levels to NAFLD remains limited [41,42,43]. Overall, although the epidemiological evidence varies across different metabolic outcomes, the accumulating evidence supports PFAS as an important environmental contributor to metabolic dysregulation, thereby providing a rationale for the mechanistic pathways discussed below.

### 2.2. Underlying Mechanism in Lipid Disturbances

#### 2.2.1. Core Dysregulation via Nuclear Receptor Signaling

Nuclear receptors (NRs) function as a vital family of transcription factors governing a broad spectrum of physiological processes, encompassing metabolism, reproduction, and inflammation [44]. These receptors act as molecular sensors for lipid metabolites, orchestrating differential gene expression programs that lead to distinct physiological outcomes. A substantial body of evidence indicates that the toxicity of PFAS is closely linked to their ability to bind and activate multiple nuclear receptors, including members of the peroxisome proliferator-activated receptor (PPAR) family, the constitutive androstane receptor (CAR), the liver X receptor (LXR), the farnesoid X receptor (FXR), and the pregnane X receptor (PXR) [45]. Notably, these receptors are central regulators of lipid homeostasis, operating through compl and interconnected signaling networks.

PPARα (also called NR1C1) is a ligand-activated nuclear receptor highly expressed in tissues characterized by high rates of fatty acid oxidation (FAO), such as the liver, brown adipose tissue, and so on [46,47,48]. A wealth of in vitro and in vivo studies, particularly those using PPARα knockout mice, have validated its biological functions. As both a nutrient sensor and transcriptional regulator, PPARα modulates hepatic metabolism and inflammatory responses by regulating the expression of genes involved in fatty acid oxidation, lipogenesis, and ketogenesis [49,50]. In recent years, PFOA, as one of the representative legacy PFAS, has been relatively well studied with respect to its mechanisms of activating PPARα. Acting as a potent exogenous ligand, PFOA initiates an adverse metabolic cascade [51,52]. A primary consequence of this activation is the transcriptional upregulation of Acyl-CoA Oxidase 1 (*Acox1*), a critical rate-limiting enzyme, which is important in lipid homeostasis as it catalyzes peroxisomal fatty acid (FA) β-oxidation [53,54]. Overactivated ACOX1 induces excessive reactive oxygen species (ROS) production in the liver, which markedly impairs mitochondrial FA β-oxidation. This phenomenon has been verified in humans, rats, and mice [54]. As a result, FAs, the natural PPAR ligands, and other lipids gradually accumulate in the liver. The intrahepatic FAaccumulation might reactivate PPARα and the downstream-related target genes to influence the hepatic lipid metabolism further [55]. Beyond ACOX1, PFOA-induced PPARα activation also dysregulates a wider gene network involved in lipid metabolism. This mainly includes the upregulation of FA oxidation genes such as Acyl-CoA Dehydrogenase Medium Chain (*Acadm*), and members of the Cytochrome P450 Family 4 Subfamily A (*Cyp4a1*-*Cyp4a3*), alongside the downregulation of key lipid metabolic genes like Apolipoprotein A1 (*Apoa1*), Apolipoprotein A5 (*Apoa5*), and Phospholipid Transfer Protein (*Pltp*) [56]. Downregulation of Apoa1 and Apoa5 impairs the transport of hepatic fatty acids to peripheral tissues, causing trapped lipids to accumulate in the liver and limiting fatty acid availability for extrahepatic metabolic processes [57,58,59,60]. In comparison, reduced Pltp expression disrupts very-low-density lipoprotein (VLDL) secretion and high-density lipoprotein (HDL) remodeling, which indirectly exacerbates hepatic lipid retention and further perturbs systemic lipid homeostasis [61,62]. Collectively, these alterations in gene expression converge to promote intracellular lipid accumulation, thereby disrupting the metabolic balance of lipids and driving the development of dyslipidemia.

Of note, studies have indicated sex-specific differences in PFOA’s regulation of certain genes. For instance, cholesterol 7α-hydroxylase (*Cyp7a1*) was specifically downregulated in male rats compared to female rats in animal studies [56]. Previous studies have shown that Cyp7a1 is a key downstream target gene of PPARα. The expression of Cyp7a1 influences cholesterol clearance and lipid accumulation because it serves as the first rate-limiting enzyme in the conversion of cholesterol to bile acids [63]. This finding may indicate differences in PFOA toxicity between genders, suggesting that subsequent studies on PFAS toxicity should take this variation into account.

Beyond PFOA, other PFAS—including legacy compounds such as PFOS and emerging alternatives such as HFPO-DA (commercial name: GenX) and 6:2 Cl-PFESA (commercial name: F-53B)—can also activate PPARα and influence lipid metabolism through highly similar mechanisms [54,64,65]. Although PPARα is highly expressed in metabolically active tissues throughout the body, the PPARα-activating effect of PFOS has been most extensively validated and consistently reproduced in the liver [66,67]. Emerging studies have reported evidence from other organs such as the intestine; nevertheless, reproducible data confirming PPARα activation by PFOS in extrahepatic tissues remain scarce on the whole [68]. Although GenX was introduced as a short-chain alternative to PFOA, studies indicate that at specific exposure concentrations, it induces stronger upregulation of PPARα and inhibits osteogenesis while promoting adipogenesis in human mesenchymal stem cells [69]. Similarly, F-53B serves as a substitute for PFOS but has been shown to upregulate PPARα more potently than PFOS at certain exposure levels [69]. This indicates that short-chain PFAS compounds are not necessarily toxicity-free, and PPARα remains a major molecular target for most PFAS congeners, while the toxicity of different PFAS may vary with carbon chain length and gender. Moreover, studies have demonstrated that GenX exhibits a faster estimated elimination rate than PFOA, whereas F-53B is eliminated far more slowly than PFOS. Therefore, when investigating the metabolic disruption induced by emerging PFAS, two key factors should be considered: their varying potency in activating PPARα and other receptors, as well as their distinct bioaccumulation capacities.

PPARγ is most highly expressed in white adipose tissue (WAT) and brown adipose tissue (BAT), where it is a master regulator of adipogenesis as well as a potent modulator of whole-body lipid metabolism and insulin sensitivity [70,71]. Research indicates that PFOA, PFOS, and HFPO-TA activate PPARγ across human, mouse, and rat species [72]. Though their maximum efficacy at PPARγ is far below that of the potent agonist rosiglitazone, suggesting that they act as partial agonists [73], the significant role of PPARγ in adipogenesis necessitates careful consideration of these compounds. Binding of the PFAS mentioned above to the receptor’s ligand-binding domain triggers a three-step activation cascade: heterodimerization with RXR, recruitment of coactivators (including *Src-1*, *Pgc-1α*, *Cbp/p300*), and binding to peroxisome proliferator response elements (PPREs). This transcriptional reprogramming upregulates genes for lipid uptake, lipogenesis, and adipokine secretion, collectively disrupting systemic lipid homeostasis [74,75]. Beyond these direct transcriptional effects, PPARγ activation by PFAS may play a critical role in directing cellular differentiation programs. Specifically, it may redirect the differentiation of bone marrow mesenchymal stem cells from the osteogenic lineage toward the adipogenic lineage, thereby promoting adipocyte formation [50,76]. This lineage shift is corroborated by in vitro studies using human mesenchymal stem cells, which focus on PFOS and PFOA. These findings demonstrate that PFOS and PFOA at environmentally relevant concentrations may enhance the expression of adipogenic markers and promote lipid droplet formation [77]. With the exception of HFPO-DA and the previously mentioned PFAS, however, the binding of other PFAS to PPARγ is considered to have low specificity and weak affinity [78]. These findings suggest that PPARγ plays a correspondingly limited role in PFAS-induced lipid metabolic toxicity.

Constitutive androstane receptor (CAR) is a key transcription factor for the xenobiotic-induced expression of various genes involved in drug metabolism and disposition, as well as hepatocarcinogenesis [79]. Notably, studies have shown that PFOA activates CAR indirectly. Current studies have confirmed that PFOA cannot directly bind to the ligand-binding domain of CAR and thus functions as an indirect activator. Its activation mechanism is distinct from the classical phenobarbital pathway; PFOA-induced CAR nuclear translocation and subsequent transcription of downstream genes do not rely on protein phosphatase 2A (PP2A) [80,81]. In addition, PFOA-mediated PPARα activation can increase cellular CAR expression, further amplifying CAR-related metabolic disturbances [81]. Nevertheless, the upstream signaling molecules initiating CAR activation by PFOA remain unclear to date.

Upon activation by PFOA, CAR promotes lipid synthesis primarily by upregulating sterol regulatory element-binding protein 1c (*Srebp-1c*), which in turn enhances the expression of lipogenic genes such as glycerol-3-phosphate acyltransferase (*Gpat*) [82,83]. Concurrently, CAR activation also suppresses lipid catabolism by downregulating carnitine palmitoyltransferase 1A (*Cpt1a*), the rate-limiting enzyme for mitochondrial FA import, thereby inhibiting β-oxidation [50,84]. Additionally, CAR induces the expression of insulin-induced gene 1 (*Insig-1*) [85]. Through a sterol-dependent mechanism, Insig-1 expression acts to inhibit cholesterol synthesis and promote the storage of cholesterol esters in hepatocytes as well [86]. Collectively, these coordinated alterations drive the development of hepatic steatosis, NAFLD, and other systemic lipid metabolic disorders. Notably, whereas the functional requirement of PPARα in PFAS-induced metabolic disruption has been comprehensively established in knockout mice, evidence from CAR-null models remains limited [87,88].

**Figure 1:**
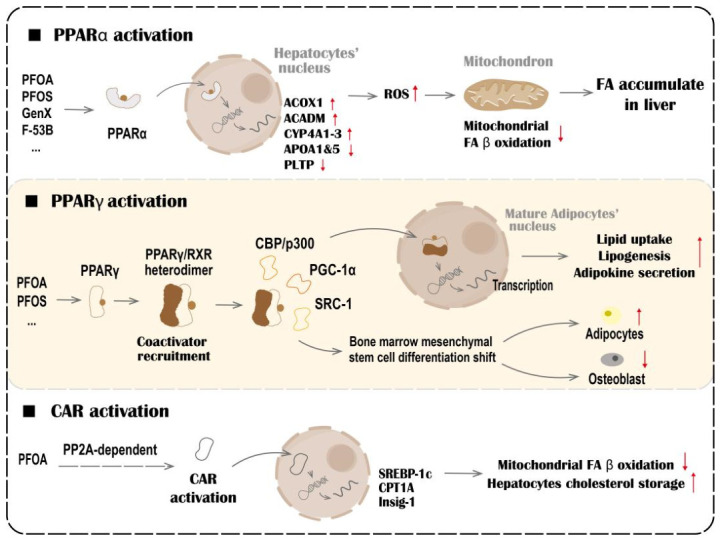
Core lipid metabolic dysregulation driven by nuclear receptor signaling activation upon PFAS exposure. Abbreviations: PPARα/γ, peroxisome proliferator-activated receptor α/γ; CAR, constitutive androstane receptor; PFAS, per- and polyfluoroalkyl substances; PFOA, perfluorooctanoic acid; PFOS, perfluorooctanesulfonic acid; RXR, retinoid X receptor; CBP/p300, PGC-1α, SRC-1, nuclear receptor coactivators; PP2A, protein phosphatase 2A; ACOX1, ACADM, CYP4A1-3, APOA1/5, PLTP, SREBP-1c, CPT1A, Insig-1, lipid metabolism-related genes; ROS, reactive oxygen species; FA, fatty acid.

Meanwhile, pregnane X receptor (PXR) and retinoid X receptor (RXR) may also play a role in PFAS-induced dysregulation. PXR is highly expressed in the liver and intestine. When activated by environmental pollutants, PXR forms a heterodimer with RXR and binds to target gene promoters, regulating genes involved in endogenous homeostasis and inflammation, thus influencing metabolic balance [89,90]. A large-scale molecular docking study has also reported a strong binding affinity for PXR. Meanwhile, toxicogenomics-related studies indicate that various PFAS can disrupt lipid metabolism through PXR [91]. These findings suggest that multiple PFAS have the potential to activate PXR and may alter metabolic homeostasis through this pathway. However, there is currently insufficient research to directly confirm the existence of this pathway, and the evidence remains very limited (Figure 1).

#### 2.2.2. Metabolic Oxidative Stress

Oxidative stress plays an important role in the toxic effects of many persistent organic pollutants (POPs). PFAS-induced metabolic disruption is also closely associated with oxidative stress, with toxicity primarily arising from mitochondrial and endoplasmic reticulum damage. Mitochondria serve as a key target for PFAS-induced toxicity, driven by multifaceted mechanisms [92]. Of note, PFAS-induced mitochondrial dysfunction occurs in multiple tissues. Aside from metabolic organs like the liver and adipose tissue, recent studies have revealed impaired oxidative phosphorylation (OXPHOS) in skeletal muscle, which is primarily attributed to mitochondrial complex I dysfunction [93]. In animal experiments, exposure to mixed PFAS has been shown to cause ultrastructural changes such as a reduction in mitochondrial cristae in lymphocytes, directly indicating impaired mitochondrial morphology [94]. Additionally, a population study has reported that prenatal exposure to PFAS, including PFOS, PFNA, and PFDeA, is associated with alterations in mitochondrial DNA copy number (mtDNAcn) in cord blood, mostly showing a decrease in mtDNAcn [95]. Another study focusing on mixed PFAS exposure in children reported this association as well [96]. These findings provide indirect evidence for PFAS-induced mitochondrial damage. Current evidence indicates that various legacy and emerging PFAS can impair mitochondrial structure (e.g., cristae reduction), disrupt mitochondrial count and membrane potential, and likely lead to excessive ROS generation through known mitochondrial mechanisms [97,98]. However, direct mechanistic research specifically linking a particular PFAS to a defined structural change in mitochondria and subsequent quantitative ROS overproduction remains relatively limited.

Comparatively, stronger evidence exists demonstrating that mitochondrial and endoplasmic reticulum damage act synergistically through disrupted iron and calcium signaling at the cellular level in vitro, thereby aggravating lipid deposition and cytotoxicity [99,100]. Specifically, PFOS upregulates acyl-CoA synthetase long-chain family member 4 (*Acsl4*), a key executor of ferroptosis, which promotes the assembly of a complex with the ER protein zinc transporter ZIP7 (Zrt-/Irt-like Protein 7), mitochondrial calcium uniporter (MCU), and mitochondrial protein voltage-dependent anion channel 3 (VDAC3) [100]. Previous studies have shown that the overexpression of *Acsl4* enriches substrates for iron-dependent lipid peroxidation on cell and mitochondrial membranes, significantly amplifying oxidative stress by promoting ferroptosis [101]. ZIP7 is involved in distributing zinc ions between the ER, cytoplasm, and mitochondria [102]; its overexpression can cause ER stress and mitochondrial dysfunction. VDAC3, as an outer membrane channel and oxidation state sensor, facilitates metabolite flux and mitochondrial ROS release but is prone to peroxidation and loss of protective function under oxidative stress [103]. Therefore, when PFOS upregulates ACSL4 and promotes its complex formation with MCU, ZIP7, and VDAC3, the combined effects of lipid substrate accumulation, metal ion imbalance, and mitochondrial ROS pathways concurrently convert normally controlled signaling ROS into uncontrolled lipid peroxidation and a ferroptosis-like amplification of oxidative stress, forming a vicious cycle of “iron overload—ROS overproduction—damage”. Currently, no studies have definitively reported that other PFAS can also activate this pathway.

**Figure 2:**
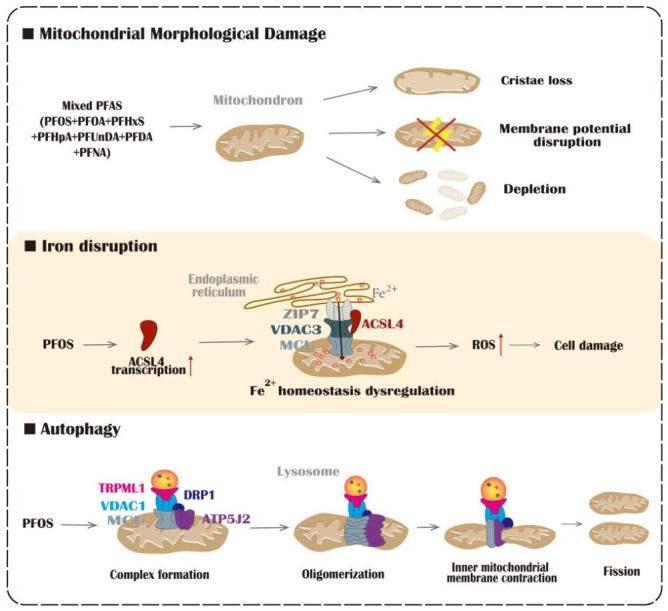
Mitochondrial oxidative stress cascades triggered by PFAS exposure. The diagram illustrates PFAS-mediated mitochondrial injury, iron homeostasis disorder and impaired autophagy that boost intracellular reactive oxygen species accumulation. Abbreviations: PFHxS, perfluorohexanesulfonic acid; PFHpA, perfluoroheptanoic acid; PFUnDA, perfluoroundecanoic acid; PFDA, perfluorodecanoic acid; PFNA, perfluorononanoic acid; ACSL4, acyl-CoA synthetase long-chain family member 4; ZIP7, zinc transporter 7; VDAC3/1, voltage-dependent anion channel 3/1; MCL1, myeloid cell leukemia 1; Fe^2+^, ferrous iron; TRPML1, transient receptor potential mucolipin 1; MCU, mitochondrial calcium uniporter; DRP1, dynamin-related protein 1; ATP5J2, ATP synthase F subunit 2.

Furthermore, relevant studies have revealed the specific mechanism by which PFOS induces mitochondrial fission in hepatocytes: it promotes the specific binding and interaction between TRPML1 on the lysosomal membrane and VDAC1 on the outer mitochondrial membrane, thereby transmitting fission signals. Meanwhile, PFOS activates the autophagy pathway by upregulating ATG5, a key autophagy gene [104], and drives the oligomerization of MCU on the inner mitochondrial membrane. The oligomerized MCU further mediates the increased expression and oligomerization of ATP5J2, a subunit of ATP synthase, and the oligomerization of ATP5J2 induces the contraction of the inner mitochondrial membrane and provides energy for fission. Finally, PFOS induces the formation of the TRPML1-VDAC1-MCU-ATP5J2 functional complex, which triggers the phosphorylation of DRP1 at the Ser616 site and recruits DRP1 to translocate to and bind to mitochondria. DRP1 then cooperates with the anchor protein FIS1 to assemble at mitochondrial fission sites, directly mediating the physical fission of mitochondria and ultimately leading to mitochondrial fragmentation and impaired energy supply (Figure 2) [92].

#### 2.2.3. Systemic Inflammation and Intertissue Communication

Systemic inflammation and intertissue communication are key processes in the toxic effects of many pollutant exposures [105]. Exposure to PFOA or PFOS alone, as well as mixed PFAS exposure, promotes the expression and secretion of pro-inflammatory cytokines such as interleukin-1β (IL-1β), interleukin-6 (IL-6), and tumor necrosis factor-α (TNF-α), while suppressing anti-inflammatory cytokines including interleukin-4 (IL-4) and interleukin-10 (IL-10), thereby disrupting immune balance [106,107]. Mechanistically, PFOA and PFOS can activate AIM2/NLRP3 inflammasomes in immune and epithelial cells [108]. The AIM2 inflammasome plays an important role in PFOS-induced inflammation. Through the Ca^2+^-PKC-NF-κB/JNK-BAX/BAK axis, PFOS causes mitochondrial DNA release and then activates AIM2 [109]. Previous studies have shown that this activation leads to the caspase-1-dependent maturation and secretion of IL-1β, as well as pyroptotic cell death [110,111]. Meanwhile, this process is accompanied by cellular disturbances such as mitochondrial DNA release, calcium dysregulation, and ER stress [112,113]. In parallel, PFOA, PFOS, and PFDS among legacy PFAS, as well as PFBS among emerging alternatives, have been shown to activate nuclear factor-kappaB (NF-κB) [114,115,116,117]. Activated NF-κB upregulates the expression of cytokines such as IL-1β and TNF-α, which in turn stimulates NF-κB, creating a self-perpetuating loop that amplifies inflammation [118,119]. These cytokines also recruit additional immune cells and stimulate further mediator release, ultimately propagating chronic inflammation and tissue injury in a domino-like cascade [120,121]. However, research on PFAS and NF-κB has primarily focused on PFOA and PFOS, with relatively limited evidence available for other PFAS.

**Figure 3:**
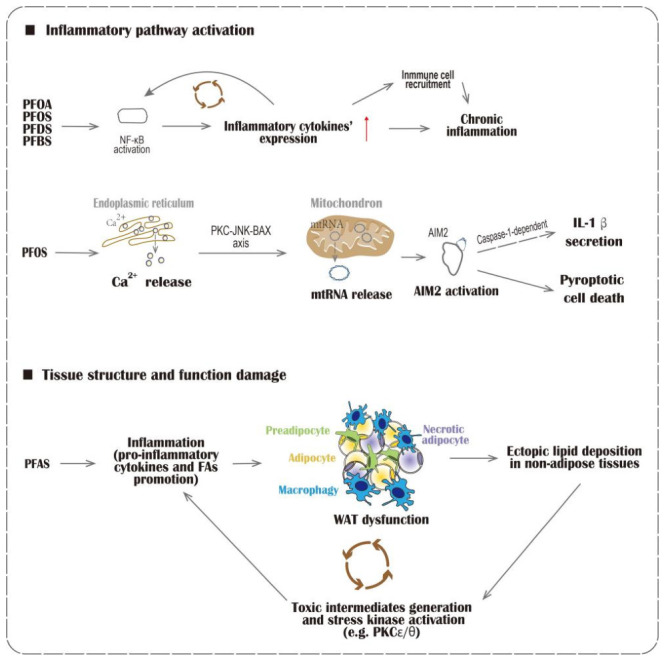
PFAS-induced chronic inflammation and disrupted inter-tissue metabolic communication. The pathway chart depicts inflammatory signaling activation and subsequent white adipose tissue dysfunction driving systemic lipid metabolic disturbance. Abbreviations: PFDS, perfluorodecanesulfonate; PFBS, perfluorobutanesulfonic acid; NF-κB, nuclear factor κ-light-chain-enhancer of activated B cells; Ca^2+^, calcium ion; PKC, protein kinase C; JNK, c-Jun N-terminal kinase; BAX, BCL-2-associated X protein; mtRNA, mitochondrial RNA; AIM2, absent in melanoma 2; IL-1β, interleukin-1β; WAT, white adipose tissue; PKCε/δ, protein kinase C epsilon/delta; FAs, fatty acids.

Moreover, they play a vital role in directly damaging tissue structure and function, especially in the liver and WAT. According to the “lipid spillover hypothesis”, dysfunctional WAT leads to ectopic deposition of free fatty acids (FFAs) in non-adipose tissues [122]. Specifically, ectopic lipid deposition leads to the generation of toxic intermediates such as diacylglycerol (DAG) and subsequent stress kinase (protein kinase C epsilon/theta, PKCε/θ) activation, ultimately impairing insulin signaling in the liver and muscle [123,124,125,126]. This process is further promoted by inflammation and ER stress [123]. Nevertheless, direct experimental evidence linking PFAS exposure to this precise systemic network remains an outstanding gap in our understanding (Figure 3).

## 3. PFAS Exposure Induces Glucose Metabolic Disorders

### 3.1. Glucose-Related Epidemiological Evidence

Cross-sectional studies first identified associations between PFAS exposure and glucose metabolism dysregulation, including exposure to PFOA, PFOS, PFHxS, and PFNA among legacy PFAS [127,128]. Existing epidemiological evidence on PFAS exposure and blood glucose or glucose metabolism disorders comes mainly from legacy long-chain PFAS, suggesting that increased attention should be paid to the potential effects of emerging PFAS on glucose metabolism. These studies mainly assessed these associations using biomarkers such as fasting plasma glucose (FPG), fasting serum insulin, or homeostatic model assessment of insulin resistance (HOMA-IR). However, these associations are not consistent across studies, with some reporting positive associations [129,130,131], while others observe null or even inverse relationships, depending on population characteristics, exposure levels, and metabolic status [132,133]. Additionally, the results are biomarker-specific [134].

Prospective cohort studies have further examined these associations, yet the findings remain heterogeneous and generally weaker than those observed in cross-sectional analyses [135,136,137]. The discrepancy between study designs may partly reflect the influence of reverse causation in cross-sectional settings, as well as differences in exposure assessment timing relative to disease development. Additionally, effect heterogeneity across PFAS congeners suggests that individual compounds may differ in their metabolic relevance, supporting the need to consider mixture-based exposure models and potential chemical interactions when interpreting epidemiological findings [138].

Growing evidence from studies focusing on vulnerable populations—such as pregnant women—suggests that prenatal PFAS exposure may adversely affect gestational glucose homeostasis and increase the risk of gestational diabetes mellitus (GDM) [127,139]. Nevertheless, findings across studies remain inconsistent, likely reflecting differences in exposure windows, population susceptibility, and outcome definitions.

Emerging studies on alternative PFAS, including Me-PFOSA-AcOH and 6:2 Cl-PFAES, have also suggested potential associations with glucose metabolism indicators [140,141], although the evidence base remains limited. Overall, current epidemiological evidence suggests possible associations between PFAS exposure and glucose metabolic dysregulation; however, substantial heterogeneity across study designs, populations, and exposure metrics precludes inference of causality.

### 3.2. Underlying Mechanism in Glucose Disturbances

#### 3.2.1. Direct Effect on Pancreatic β-Cell Function and Insulin Signaling

Evidence suggests that PFOA and PFOS exert direct toxic effects on the pancreas, particularly on insulin-producing β-cells [142,143]. Insulin biosynthesis is governed by a network of transcription factors, such as pancreatic and duodenal homeobox 1 (*Pdx-1*) and insulin enhancer-binding protein 1 (*Islet-1*) [144]. Specifically, *Pdx-1* is a key activator of insulin gene transcription and also regulates β-cell survival and function [145], while *Islet-1* acts as an upstream regulator in maintaining the β-cell transcriptional program [146]. Exposure to PFOS has been shown to significantly reduce both protein and mRNA expression of *Pdx-1* and *Islet-1* in pancreatic islets or β-cell lines [147], indicating that PFOS can directly suppress insulin synthesis by disrupting transcriptional regulation in β-cells. This line of evidence integrates a key element into the pathophysiology of PFAS-induced glucose dysregulation by suggesting that, alongside peripheral insulin resistance, a concurrent role for impaired insulin secretion must be considered.

The insulin signaling pathway serves as the central hub for glucose homeostasis, and its dysregulation at any step can lead to insulin resistance [148]. Both in vivo and in vitro evidence indicates that PFAS, particularly long-chain compounds such as PFOA and PFOS, can interfere with the normal function of this pathway. Under normal physiology, insulin action is initiated by its binding to the membrane receptor (INSR), triggering tyrosine phosphorylation of the intracellular β-subunit. This subsequently recruits and phosphorylates insulin receptor substrates to form the key signaling platform [149]. Within insulin signaling, the phosphatidylinositol 3-kinase (PI3K)/protein kinase B (AKT) pathway serves as the central hub regulating glucose metabolism [150]. Insulin binding activates PI3K, which catalyzes the generation of phosphatidylinositol 3,4,5-trisphosphate (PIP3) [151,152]. This second messenger then recruits and activates AKT. Activated AKT phosphorylates downstream substrates such as glycogen synthase kinase 3β (GSK3β) [153,154], promoting hepatic glycogen synthesis and driving the translocation of glucose transporter type 4 (GLUT4) to the cell membrane [155]. This process mediates glucose uptake in both skeletal muscle and adipocytes, thus maintaining blood glucose homeostasis. However, substantial evidence shows that PFAS like PFOA potently inhibit this pathway by upregulating PTEN and suppressing AKT/GSK3β phosphorylation, thereby attenuating insulin signaling, reducing GLUT4 translocation, and impairing glucose uptake [156,157]. Mechanistically, these effects may stem from dampened insulin receptor signaling or from PFOA-induced lipotoxicity/oxidative stress. Together, these findings highlight the inhibition of the PI3K/AKT axis as a central mechanism contributing to systemic insulin resistance.

**Figure 4:**
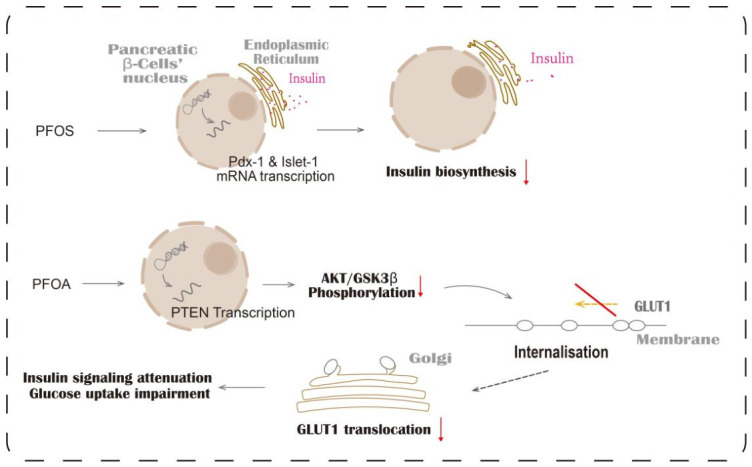
PFAS impairs insulin biosynthesis in pancreatic β-cells and GLUT1-dependent peripheral glucose uptake. Abbreviations: Pdx-1, pancreatic and duodenal homeobox 1; Islet-1, insulin gene enhancer protein ISL-1; PTEN, phosphatase and tensin homolog; AKT, protein kinase B; GSK3β, glycogen synthase kinase 3β; GLUT1, glucose transporter 1.

In addition, the inhibition of AKT triggers crosstalk with other vital pathways involved in energy homeostasis. AMP-activated protein kinase (AMPK), a canonical energy-sensing pathway antagonistic to AKT, exhibits increased activity when AKT signaling is suppressed [158,159]. Altered AKT activity also modulates the function of PPARs, especially PPARγ, which is elaborated on in the following section [160,161]. This interactive network jointly disrupts glucose and lipid metabolism and aggravates PFAS-induced insulin resistance (Figure 4).

#### 3.2.2. PPARs Signaling Dysregulation

As mentioned in the section on lipid metabolism, PFAS act as established agonists of PPARs, with PPARα activation representing a well-established molecular mechanism [162]. As exogenous toxicants, PFOS, PFOA, and other PFAS can induce sustained activation of PPARs, disrupting glucose metabolic homeostasis and contributing directly to glucose metabolism disorders through several interconnected mechanisms. First and importantly, robust PPARα activation upregulates key enzymes in the peroxisomal β-oxidation pathway, such as ACOX1 mentioned above, thereby exacerbating oxidative stress [54]. Second, the aberrant transcriptional programs driven by PFOA-activated PPARs disturb the balance between lipid and glucose metabolism, as evidenced by PPARα-dependent induction of hepatic steatosis and NAFLD in animal studies [42]. In addition to PPARα, other receptors also play a role in this process. Animal studies have shown that even under conditions of PPARα silencing, PFOA exposure can still activate and upregulate CAR to influence related physiological effects, suggesting a multi-targeted capacity for metabolic disruption [162]. Collectively, these upstream disruptions converge to impair the central insulin-signaling PI3K–AKT pathway. Evidence from in vivo and HepG2 cell models shows that PFOS exposure suppresses PTEN expression. This suppression leads in turn to the dysregulation of the PI3K–AKT signaling pathway [163,164,165]. Given the important role of AKT in cellular insulin sensitivity, its inhibition promotes hepatic gluconeogenesis and insulin resistance. Currently, direct evidence linking PFAS to AKT-dependent disruption remains confined to PFOA and PFOS, with data on other congeners being limited [165,166]. Importantly, the applicability of these findings to human exposure scenarios requires careful evaluation, as some studies suggest NR activation may be secondary at human-relevant exposures. For instance, one study reported that they observed significant activation only when exposed to PFOA, PFOS, and PMOH at concentrations that were much higher than typical human exposure levels (>10 μM) [167], highlighting the need for research at physiologically relevant doses to clarify the primary drivers of toxicity.

#### 3.2.3. Oxidative Stress and Inflammatory Activation

Oxidative stress and chronic low-grade inflammation represent two other critical drivers of insulin resistance. In the process by which PFAS disrupt glucose metabolism, oxidative stress and inflammation also play a significant role. As previously outlined, PFAS not only reduce ATP production but also cause excessive ROS generation by disrupting mitochondrial iron homeostasis and activating the PPARα-ACOX1 axis to enhance peroxisomal β-oxidation. PFOA and PFOS also induce granulosa cell apoptosis via the SIRT1/FOXO1-SOD2 pathway, which further impairs mitochondrial antioxidant capacity [72]. ROS further disrupt insulin signaling through multiple cytotoxic pathways, including the oxidation of polyunsaturated fatty acids, disruption of redox balance, enzyme inactivation, DNA damage, promotion of membrane lipid peroxidation, and increased malondialdehyde levels [168,169]. Research is ongoing to clarify the upstream mechanisms of PFAS-induced mitochondrial dysfunction. For instance, a 2023 cell-based study reported that PFOS exposure triggers hepatic insulin resistance by inducing mitochondrial iron overload, mediated by transferrin receptor 2 (TFR2) and ATP synthase β subunit (ATP5B) [170]. However, the precise underlying mechanisms still require further elucidation.

Recently, emerging studies have also identified substantial impairments in mitochondrial oxidative phosphorylation in skeletal muscle following PFAS exposure [93]. Given the established role of mitochondrial function in maintaining cellular iron homeostasis, impaired oxidative phosphorylation may also contribute to iron dyshomeostasis in skeletal muscle, although direct experimental evidence remains limited [171,172]. Such mitochondrial abnormalities are therefore likely to exacerbate insulin resistance by further compromising metabolic homeostasis across multiple tissues.

**Figure 5:**
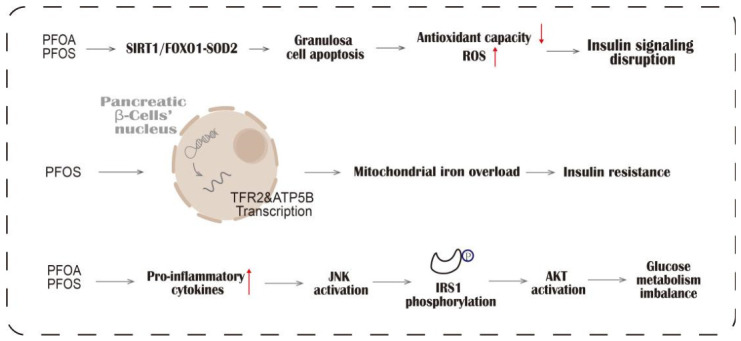
Oxidative stress and inflammatory signaling pathways driving PFAS-induced insulin resistance. Abbreviations: SIRT1, sirtuin 1; FOXO1, forkhead box O1; SOD2, superoxide dismutase 2; TFR2, transferrin receptor 2; ATP5B, ATP synthase subunit beta; IRS1, insulin receptor substrate 1.

Chronic inflammation is another vital driver of insulin resistance. As noted earlier, PFAS such as PFOA and PFOS promote the expression and secretion of pro-inflammatory cytokines (e.g., TNF-α, IL-6) and activate canonical inflammatory pathways, including NF-κB and JNK [173]. Activated JNK directly phosphorylates IRS-1 serine residues (e.g., Ser307), thereby promoting IRS-1 tyrosine phosphorylation and its binding to AKT and disrupting glucose metabolism balance [174]. Similarly, NF-κB activation promotes insulin resistance both by inducing cytokine expression and potentially interacting directly with insulin signaling molecules [175]. Studies show that JNK inhibitors block PFOS-induced inflammation, clearly defining the causal chain: PFOS exposure → pro-inflammatory cytokine release → JNK activation → IRS-1 serine phosphorylation → insulin resistance. Thus, PFAS-triggered inflammatory cascades constitute a critical bridge linking environmental exposure to systemic metabolic dysfunction (Figure 5).

#### 3.2.4. Gut Microbiota Alterations

**Figure 6:**
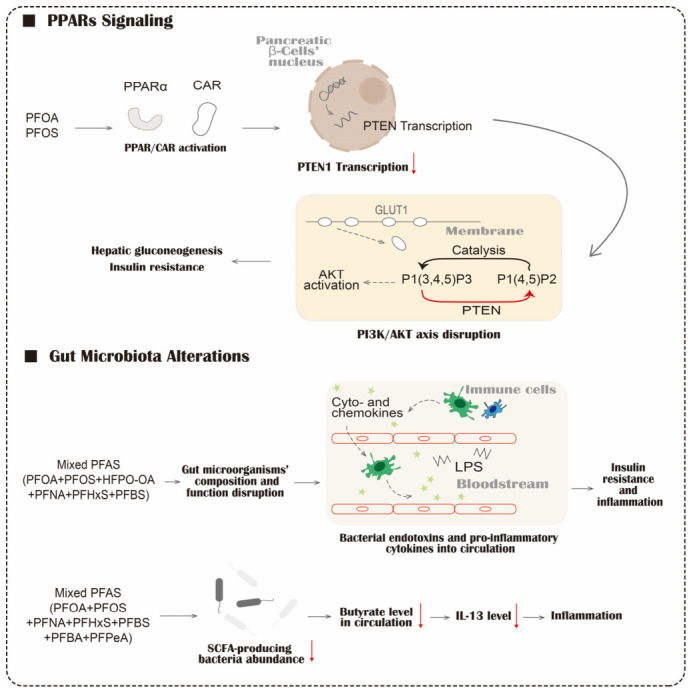
PPARs and gut microbiota dysbiosis mediate PFAS-related glucose metabolic disorders.

Growing evidence highlights the gut microbiota as a critical intermediary in environmental pollutant toxicity. An in vitro study reported that PFAS co-exposure (containing PFOA, PFOS, PFBS, PFNA, PFHxS, and HFPO-DA) alters both the composition and functional profile of gut microorganisms in a human colonic model [176]. A primary consequence of this dysbiosis is the impairment of intestinal barrier function, which facilitates the translocation of bacterial endotoxins such as lipopolysaccharide (LPS) into the portal circulation [177]. Once in the liver, LPS activates immune cells and exacerbates hepatic inflammation and insulin resistance as previously described. Additionally, microbial disturbances alter bile acid metabolism. Acting as signaling molecules, bile acids regulate glucose and lipid homeostasis through receptors such as FXR and the G protein-coupled bile acid receptor 1 (GPBAR1, also known as TGR5) [178]. This dysbiosis may alter the bile acid pool, thereby disrupting these signaling pathways [26]. Emerging evidence from studies on maternal (including PFOA, PFOS, PFHxS, PFNA, PFBA, PFPeA, and PFBS) exposure suggests that this mechanism may also operate in offspring, pointing to potential intergenerational effects [179]. SCFAs (e.g., acetate, propionate, and butyrate) are produced by microbial fermentation of dietary fiber and serve as both energy substrates and signaling molecules [180,181]. SCFAs stimulate intestinal L cells to secrete glucagon-like peptide-1 (GLP-1), a hormone that promotes insulin secretion in a glucose-dependent manner, delays gastric emptying, and enhances satiety [182]. Studies indicate that PFAS co-exposure may reduce the abundance of SCFA-producing bacteria, potentially lowering circulating butyrate levels. This decline could weaken butyrate’s positive feedback on the anti-inflammatory cytokine IL-13, thereby influencing systemic inflammation [176]. However, it should be noted that some studies have reported contradictory findings, including elevated SCFA levels following PFAS exposure [183], highlighting the need for further high-quality research to clarify these associations (Figure 6).

## 4. Discussion

### 4.1. Integrated Mechanisms Underlying PFAS-Disturbed Glucose and Lipid Metabolic Homeostasis

Based on the current body of evidence, mitochondrial dysfunction appears to represent one of the earliest and most sensitive events following PFAS exposure. Mitochondrial damage and oxidative stress are prominent initiating events during PFAS-induced metabolic toxicity. Impaired oxidative phosphorylation and disrupted cellular iron homeostasis occur across different tissues, and these primary alterations sequentially disturb downstream pathways governing glucose and lipid metabolism, including the PI3K/AKT axis, AMPK, and PPAR signaling. Rather than acting independently, these pathways are extensively interconnected, with mitochondrial dysfunction serving as a central hub that both initiates and amplifies disturbances in insulin signaling, inflammatory responses, and lipid metabolism.

Building upon this integrated framework, PFAS-mediated metabolic disruption can be conceptualized as four interconnected pathological modules rather than independent molecular events. First, PFAS function as exogenous disruptors of key metabolic sensors, primarily by hijacking nuclear receptor signaling. Through sustained and often aberrant activation of receptors such as PPARα, PPARγ, PXR, and CAR, PFAS coordinately reprogram transcriptional networks governing lipogenesis, fatty acid oxidation, and insulin sensitivity across major metabolic tissues. This receptor-mediated reprogramming sets a coherent, yet pathological, metabolic tone that simultaneously predisposes individuals to hepatic steatosis, adipose tissue dysfunction, and systemic insulin resistance.

Second, PFAS induce integrated stress across critical cellular organelles, most notably in the mitochondria and the endoplasmic reticulum. This insult manifests as compromised energy metabolism characterized by impaired fatty acid β-oxidation and mitochondrial OXPHOS, excessive generation of ROS, and calcium dyshomeostasis. Rather than being confined, these stress signals function as ubiquitous drivers that impair insulin signal transduction, activate pro-inflammatory kinases, and trigger cell death pathways across target tissues, thereby creating a shared cellular basis for diverse metabolic defects.

The damage signals emanating from stressed organelles directly feed into a third, potent trigger: the activation of a persistent, low-grade inflammatory state. PFAS promote inflammasome assembly and the release of cytokines (e.g., TNF-α, IL-1β, IL-6) from immune and metabolic cells. These inflammatory mediators, through pathways such as JNK and NF-κB, directly inhibit insulin action via serine phosphorylation of IRS proteins and further aggravate organelle stress, establishing a vicious cycle that perpetuates and amplifies metabolic resistance.

Finally, the molecular disturbances initiated by the above triggers reconfigure the essential physiological dialogues between metabolic organs. The normal cross-talk among the liver, adipose tissue, skeletal muscle, and the gut is subverted into a pathological network [184,185,186]. The “lipid spillover” from dysfunctional adipose tissue, the disrupted gut-liver axis mediated by dysbiosis and endotoxin translocation, and the endocrine release of hepatokines (e.g., fetuin-A) from a fatty liver exemplify how localized cellular insults are translated into integrated systemic disease [187,188].

Importantly, while mitochondrial dysfunction may represent an early initiating event, the downstream pathological modules do not operate linearly but instead engage in extensive cross-talk, forming a dynamic, self-reinforcing toxicity network. For instance, mitochondrial ROS can activate inflammatory pathways, which in turn exacerbate ER stress and further dysregulate nuclear receptor activity. This non-linear interplay means that the systemic metabolic outcome is greater than the sum of individual pathway disruptions. Such synergistic effects further amplify metabolic dysfunction and accelerate the progression of hepatic steatosis, insulin resistance, and other related metabolic phenotypes. In addition, a wealth of epidemiological and toxicological evidence demonstrates that PFAS exposure disrupts metabolic homeostasis across multiple organ systems, including the brain, cardiovascular system, hematopoietic tissue, and reproductive organs [40,99,189,190,191,192,193]. Such broad systemic metabolic disturbances associated with PFAS warrant comprehensive collation and analysis in an independent, dedicated review.

### 4.2. Research Limitations and Future Computational and Technological Perspectives

Current evidence linking PFAS exposure to lipid and glucose metabolism has important limitations. First, metabolic toxicity varies substantially across PFAS congeners. Many novel PFAS alternatives exert PPAR regulatory effects equal to or more pronounced than those of legacy PFAS. Given their environmental persistence and ongoing industrial emissions, alternative PFAS may impose long-term latent metabolic risks that remain insufficiently characterized. Second, although the metabolic effects of well-known PFAS such as PFOA and PFOS on lipid and glucose homeostasis have increasingly reached consensus, inconsistencies still exist across studies, and the comprehensive and in-depth toxic mechanisms remain far from fully clarified. Three key factors account for inconsistent results: differences in the specific PFAS congeners investigated; variations in exposure concentrations, timing, and experimental durations, such as differing follow-up periods in cohort studies; and the fact that lipid and glucose metabolism are jointly affected by environmental and genetic conditions. We have not yet fully clarified how factors such as race, gender, age, metabolic status, and genetic background modify toxic outcomes, let alone the inherent difficulty in accurately assessing actual external human exposure levels. All these issues show that more high-quality long-term population studies are urgently needed. Third, most mechanism data come from standard cell and animal experiments. These models have physiological differences from humans, so we cannot easily extend their findings to human metabolic diseases. This means we need to use more human-relevant test systems such as organoids, which better simulate whole-body human biological interactions. Furthermore, current mechanistic investigations are largely limited to the liver, representing a substantial organ-specific bias. Taken together, we still have a long way to go in fully understanding the disruptive effects of PFAS on lipid and glucose metabolism.

To address the aforementioned drawbacks restricting PFAS metabolic research, wearable monitoring tools and advanced machine learning frameworks can be introduced to advance refined PFAS exposure measurement and predictive assessment of metabolic toxic mechanisms.

Wearable biosensors, such as nanosensors, can overcome the limitation of one-off cross-sectional blood testing, enabling long-term non-invasive tracking of continuous personal PFAS exposure. Such devices can synchronously capture dynamic glycemic and lipid biomarkers, establishing real-time links between chronic pollutant exposure and metabolic fluctuations [194,195]. Combined with spatial environmental monitoring data, these tools also support population-level mapping of PFAS pollution and related metabolic disease risks [195,196].

Machine learning complements traditional linear statistics and laboratory experiments. Interpretable algorithms can disentangle non-linear mixture interactions among diverse PFAS congeners, explaining inconsistent epidemiological observations across cohorts. AI-assisted multi-omics profiling enables researchers to pinpoint core lipid and metabolic molecular signatures disrupted by PFAS exposure [56,197]. In parallel, quantitative structure–activity relationship (QSAR) and molecular docking models offer valuable computational support for toxicological research. These tools can swiftly characterize binding interactions between newly manufactured PFAS alternatives and metabolism-associated protein targets, select promising protein candidates for subsequent laboratory validation, and assist in deducing underlying toxic pathways when direct experimental toxicological evidence is scarce [198,199].

Despite promising prospects, these technical approaches still have limitations: most predictive models lack multi-cohort validation, and computational outputs and wearable monitoring data need experimental verification. Collectively, combining wearable dynamic exposure tracking and interpretable computational modeling will reconcile contradictory evidence, clarify core pathways of PFAS-induced glucose and lipid dysfunction, and facilitate accurate risk assessment for exposed populations.

## 5. Conclusions

PFAS exposure has emerged as an important environmental contributor to metabolic disorders by disrupting the coordinated regulation of lipid and glucose homeostasis. Rather than affecting individual metabolic pathways independently, accumulating evidence indicates that PFAS-induced alterations in lipid and glucose metabolism are interconnected through shared molecular mechanisms, which may collectively accelerate metabolic dysfunction and increase susceptibility to chronic metabolic diseases. However, inconsistencies among epidemiological findings, species-specific differences between experimental models and humans, and the complexity of mixed PFAS exposure continue to limit a comprehensive understanding of the causal relationship between PFAS exposure and metabolic disorders.

Future studies integrating longitudinal epidemiological investigations with mechanistic research, multi-omics analyses, and emerging computational approaches will be essential to resolve these uncertainties and improve the translation of experimental findings into human health risk assessment. A more comprehensive understanding of PFAS-associated metabolic dysregulation will not only facilitate the identification of potential therapeutic targets but also provide stronger scientific evidence for PFAS risk assessment and regulatory decision making.

## Figures and Tables

**Table 1:** Major per- and polyfluoroalkyl substances (PFAS) discussed in this review. Condensed structural formulas are included for reference. PFAS nomenclature complies with guidelines from ITRC and ATSDR, as well as the standard terminology proposed by Buck et al. [2].

Compound	Abbreviation	Condensed Formula
**Perfluoroalkyl substances**		
*Perfluoroalkyl carboxylic acids (PFCAs)*		
Perfluorobutanoic acid	PFBA	CF_3_(CF_2_)_2_COOH
Perfluorononanoic acid	PFOA	CF_3_(CF_2_)_6_COOH
Perfluorodecanoic acid	PFNA	CF_3_(CF_2_)_7_COOH
Perfluorooctanoic acid	PFDA	CF_3_(CF_2_)_8_COOH
*Perfluoroalkane sulfonic acids (PFSAs)*		
Perfluorobutane sulfonic acid	PFBS	CF_3_(CF_2_)_3_SO_3_H
Perfluorohexane sulfonic acid	PFHxS	CF_3_(CF_2_)_5_SO_3_H
Perfluorooctane sulfonic acid	PFOS	CF_3_(CF_2_)_7_SO_3_H
**Polyfluoroalkyl substances**		
*Perfluoroalkane sulfonamido derivatives*		
2-(N-methyl-perfluorooctane sulfonamido)acetic acid	MeFOSAA	CF_3_(CF_2_)_7_SO_2_
		-N(CH_3_)CH_2_COOH
**Emerging/alternatives PFAS**		
*Chlorinated polyfluoroalkyl ether sulfonates*		
6:2 Chlorinated polyfluoroalkyl ether sulfonate	6:2 Cl-PFAES	Cl(CF_2_)_6_O(CF_2_)_2_SO_3_H
6:2 Chlorinated polyfluorinated ether sulfonate	F-53B	Cl(CF_2_)_3_O(CF_2_)_5_SO_3_H
*Hexafluoropropylene oxide (HFPO)-derived carboxylic acids*		
2,3,3,3-Tetrafluoro-2-(heptafluoropropoxy)propanoic acid	GenX	CF_3_CF(CF_3_)OCF(CF_3_)COOH
Hexafluoropropylene oxide trimer acid	HFPO-TA	CF_3_CF(CF_3_)O−CF(CF_3_)OCF(CF_3_)COOH
*Other alternative polyfluoroalkyl substances*		
Perfluoro-2-methoxyhexanoic acid	PFMOHA	CF_3_(CF_2_)_3_CF(OCH_3_)−COOH

## Data Availability

No new data were created or analyzed in this study. Data sharing is not applicable to this article.

## Abbreviations

6:2 Cl-PFAES	6:2 Chlorinated polyfluoroalkyl ether sulfonate
ACADM	Acyl-CoA Dehydrogenase Medium Chain
ACOX1	Acyl-CoA Oxidase 1
ACSL4	Acyl-CoA synthetase long-chain family member 4
Akt	Protein kinase B
AMPK	AMP-activated protein kinase
APOA	Apolipoprotein A
ATP5B	ATP synthase β subunit
CAR	The constitutive androstane receptor
CPT1A	Carnitine palmitoyltransferase 1A
CYP4A	Cytochrome P450 Family 4 Subfamily A
DAG	Diacylglycerol
F-53B	6:2 Chlorinated polyfluorinated ether sulfonate
FA	Fatty acid
FAO	Fatty acid oxidation
FFAs	Free fatty acids
FPG	Fasting plasma glucose
FXR	The farnesoid X receptor
GDM	Gestational diabetes mellitus
GenX	2,3,3,3-Tetrafluoro-2-(heptafluoropropoxy)propanoic acid
GLP-1	Glucagon-like peptide-1
GLUT4	Glucose transporter type 4
GPAT	Glycerol-3-phosphate acyltransferase
GPBAR1	G protein-coupled bile acid receptor 1
GSK3β	Glycogen synthase kinase 3β
HDL-C	High-density lipoprotein cholesterol
HFPO-TA	Hexafluoropropylene oxide trimer acid
HOMA-IR	Homeostatic model assessment of insulin resistance
IDF	The International Diabetes Federation
IL-10	Interleukin-10
IL-1β	Interleukin-1β
IL-4	Interleukin-4
IL-6	Interleukin-6
Insig-1	Insulin-induced gene 1
InsR	Insulin receptor
Islet-1	Insulin enhancer-binding protein 1
LDL-C	Low-density lipoprotein cholesterol
LPS	Lipopolysaccharide
LXR	The liver X receptor
MCU	Mitochondrial calcium uniporter
Me-PFOSA-AcOH	2-(N-methyl-perfluorooctane sulfonamido)acetic acid
mtDNAcn	Mitochondrial DNA copy number
NAFLD	Non-alcoholic fatty liver disease
NF-κB	Nuclear factor-kappaB
NRs	Nuclear receptors
OXPHOS	Oxidative phosphorylation
Pdx-1	Pancreatic and duodenal homeobox 1
PFAS	Per- and polyfluoroalkyl substances
PFBA	Perfluorobutyric acid
PFBS	Perfluorobutanesulfonic acid
PFCAs	Perfluoroalkyl carboxylic acids
PFDeA	Perfluorodecanoic acid
PFHxS	Perfluorohexane sulfonic acid
PFNA	Perfluorononanoic acid
PFOA	Perfluorooctanoic acid
PFOS	Perfluorooctane sulfonic acid
PFSAs	Perfluoroalkane sulfonic acids
PI3K	Phosphatidylinositol 3-kinase
PIP3	Phosphatidylinositol 3,4,5-trisphosphate
PLTP	Phospholipid Transfer Protein
PMOH	Perfluoro-2-methoxyhexanoic acid
POPs	Persistent organic pollutants
PPAR	The peroxisome proliferator-activated receptor
PPREs	Peroxisome proliferator response elements
PXR	The pregnane X receptor
ROS	Reactive oxygen species
RXR	Retinoid X receptor
SREBP-1c	Sterol regulatory element-binding protein 1c
T2DM	Type 2 diabetes mellitus
TC	Total cholesterol
TFR2	Transferrin receptor 2
TNF-α	Tumor necrosis factor-α
VDAC3	Voltage-dependent anion channel 3
WAT	White adipose tissue
ZIP7	Zrt-/Irt-like Protein 7
